# Editorial: International Plant Proteomics Organization (INPPO) World Congress 2014

**DOI:** 10.3389/fpls.2016.01190

**Published:** 2016-08-05

**Authors:** Joshua L. Heazlewood, Jesús V. Jorrín-Novo, Ganesh K. Agrawal, Silvia Mazzuca, Sabine Lüthje

**Affiliations:** ^1^Lawrence Berkeley National Laboratory, Physical Biosciences Division, Joint BioEnergy InstituteBerkeley, CA, USA; ^2^Australian Research Council Centre of Excellence in Plant Cell Walls, School of BioSciences, The University of MelbourneMelbourne, VIC, Australia; ^3^Agricultural and Plant Biochemistry and Proteomics Research Group, Department of Biochemistry and Molecular Biology, University of CordobaCordoba, Spain; ^4^Research Laboratory for Biotechnology and BiochemistryKathmandu, Nepal; ^5^Global Research Arch for Developing Education Academy Private LimitedBirgunj, Nepal; ^6^Laboratorio di Biologia e Proteomica Vegetale, Dipartimento di Chimica e Tecnologie Chimiche, Università della CalabriaRende, Italy; ^7^Oxidative Stress and Plant Proteomics Group, Biocenter Klein Flottbek and Botanical Garden, University of HamburgHamburg, Germany

**Keywords:** plant proteomics, mass spectrometry, 2-DE, world congress

The discipline of proteomics has undergone considerably advances over the past two decades. Our ability to delve deeper into complex proteomes, identify post-translational modifications, and profile protein abundance has greatly expanded the utilization of mass spectrometry in biology. The plant research community has enthusiastically embraced proteomic approaches and has applied these technologies to explore a multitude of research questions in the field of plant biology (Jorrín-Novo et al., 2015). In 2011, a group of plant proteomic researchers established the International Plant Proteomics Organization (INPPO) to advance the application of this technology in plants and agriculture (Agrawal et al., 2011). The INPPO conducted its inaugural world congress in the autumn of 2014 at the University of Hamburg (Germany) (Lüthje et al., 2015). The meeting brought together leading international experts in plant proteomics and provided a critical mass for the discussion of proteomic technologies and their application in all aspects of plant biology. This Research Topic arose from this meeting as a means to capture current research, views, and approaches from the wider plant proteomics community.

## Technical advances in plant proteomics

As the field of proteomics has evolved, many analytical approaches have relied on advancements in instrumentation as well as the progression of techniques to exploit these changes. Accordingly, this Research Topic highlights a range of updated approaches and provides a number of viewpoints on current technical limitations that are especially pertinent to plant proteomics researchers. Often it is necessary for plant researchers to adapt approaches or push techniques and concepts developed in other species into the field of plant biology. The development of proteomics standards or MIAPEs (Minimum Information About a Proteomics Experiment) were developed by the mammalian field as part of the Proteomics Standards Initiative (Taylor et al., 2007). The strict adoption of these reporting guidelines vary significantly amongst journals and there are various opinions in the plant community as whether they should be strictly adopted. However, in order to make valuable contributions to the proteomics field as a whole, the plant proteomics community should be more willing to embrace these guidelines (Jorrin Novo).

### Two-dimensional gel electrophoresis-based

The limited research dollars in plant biology has resulted in the persistence of older proteomic technologies. The approach of arraying and quantifying samples by 2-DE has been employed since the 1970s (O'farrell, 1975), however the past decade has seen the adoption of gel-free or shotgun approaches dominating the field. Thus, it is not surprising that many feel it is time to reassess the dominance of 2-DE in plant biology (Anguraj Vadivel), albeit with a view to synergy rather than completely exorcizing the past. Nonetheless, submissions employing and examining new ways to exploit 2-DE were still a common theme. Adaptations to the standard separation protocols where a non-reducing first dimension coupled to a clear native electrophoresis in the second dimension highlighted a means to produce phos-tag zymograms, enabling the in-gel detection of protein phosphorylation (Meisrimler et al.). The technical and laborious nature of standard 2-DE has seen researchers explore the potential of small scale 2-DE using reduced immobilized pH gradient strips (7 cm) and precast mini-gels to identify relevant proteins in a complex lysate, namely from wheat grain extracts (Fekecsova et al.). The success of the approach is highly relevant to plant science given its use of 2-DE and could enable high throughput screens due to its simplicity and reduced sample requirements. A major limitation with 2DE (and to a lesser degree with gel-free approaches) is the inability to survey deep into the total proteome. The hunt for low-abundance proteins from complex plant samples requires the depletion of abundant plant-specific polypeptides such as RuBisCO which is highly abundant in photosynthetic material to enable the visualization of low abundance proteins (Gupta et al.).

In recent years, most phosphoproteomic studies have been conducted using gel-free or shotgun approaches with enriched samples. However, historically the use of 2-DE was seen to hold some advantages for the analysis of phosphorylation, least of which was the visual change in isoelectric point of a protein when its phosphorylation state changed. The advent of sensitive fluorescent dye for phosphorylation (Pro-Q), has more readily enabled the visualization of phosphoproteins by 2-DE. To advance this approach to enable simultaneous protein quantification, a multiplexed approach employing Pro-Q, and the fluorescent protein dye SYPRO Ruby was undertaken on germinating seeds and seedlings of a non-orthodox plant species, *Quercus ilex* (Romero-Rodriguez et al.). The approach was capable of simultaneously determining both protein changes and phosphorylation changes from samples.

### Mass spectrometry-based

The past decade has seen major advances in instrumentation used for proteomic analyses. This has included improvements in sample delivery systems (e.g., nanoflow Ultra-High Performance Liquid Chromatography), mass analyzer (e.g., orbitrap mass analyzer) and fragmentation methods (e.g., electron-transfer dissociation). Collectively these advances have improved sensitivity, dynamic range, and mass resolution which have dramatically improved our capacity to identify and characterize proteins (Heazlewood, 2011). While developments in instrumentation have contributed significantly to advancing the field, there are still applications for “older” technologies such as MALDI-ToF. These instruments were at the forefront of the proteomics revolution, but in the past decade their use has diminished. However, they are still manufactured, relatively simple to use and can be acquired at a reasonable cost. Consequently, they have gained traction for their ability to easily profile samples, discriminate between species and identify pathogens through specific biomarker profiling (Mehta and Silva). These approaches are only now being applied for species determination in plants, but could also be used to assist with breeding and plant biotechnology.

Subcellular proteomics seeks to determine the functions and constituents of organelles, complexes and compartments within the plant cell. Insight into the biology of these systems is often hampered by technical limitations associated with subcellular enrichment strategies. The assessment of organelle purity is usually undertaken by immunoblotting; however, the field of plant biochemistry is significantly restricted by the lack of commercial antibodies that can be used to assess contamination. Targeted proteomic approaches (multiple reaction monitoring) provides an alternative approach to assess the relative abundance of organelle marker proteins in a plant lysate (Parsons and Heazlewood).

The biosynthesis of *N*-glycans is a well-characterized pathway of plant endomembrane with nearly all biosynthesis members elegantly characterized by molecular genetics techniques. In plants, few studies have applied advanced mass spectrometry-based approaches to survey *N*-glycans. Thus far, only the characterization of sites harboring *N*-glycans have been undertaken, with no analysis of site specific *N*-glycan heterogeneity occurring. It has recently been shown that combinatorial fragmentation approaches involving electron-transfer dissociation (ETD) and higher-energy collisional dissociation (HCD) are capable of being used to identify and characterize *N*-glycopeptides. This technique has been highlighted on a single plant *N*-glycopeptide (Ford et al.) and the authors further highlight how advances to this approach can be used to characterize this post-translational modification in complex protein lysates.

### Sample-based

Sample preparation is at the core of all proteome surveys in plant science. The advanced analytical capabilities found in current mass spectrometers are of little value if sample integrity is compromised. Pertinent examples are surveys of plant subcellular proteomes, where sample preparation is much more important than any downstream characterization. The cell wall proteome is a unique compartment in plants and can be difficult to enrich from contamination cellular proteins. Thus, it is not surprising that the utilization of sequential extraction procedures involving CaCl_2_, EGTA, and LiCl-complemented buffers is crucial to achieve adequate enrichment (Printz et al.). Such intricate sample preparation procedures represent one of the only ways to confidently assign subcellular locations to a protein identified through proteomic characterizations.

Efforts to survey the entire proteome are significantly restricted by the limitations of current instruments to handle the sample complexity found in higher eukaryotes. This complexity is exemplified by the analysis of protein phosphorylation, an often difficult post-translational modification to confidently detect and quantify. While various phosphoproteomic techniques have been developed, progress in sample enrichment has been at the forefront of enabling reliable phosphoproteomic surveys. Many of these advances, as applied to plant biology have been summarized (Li et al.); the requirement for phosphopeptide and/or phosphoprotein enrichment is central to these highlighted approaches. A case in point is the analysis of signal transduction by mitogen-activated protein kinases (MAPKs). Profiling the phosphoproteomes of transgenic plants with manipulated MAPK pathways is at the forefront of investigations into dissecting these signaling pathways (Takáč and Šamaj). Such approaches require reproducible enrichment strategies and reliable phosphopeptide assignments and quantification procedures.

The manipulation of samples to enable reproducible quantification has been a feature of proteomics and include peptide labeling (e.g., iTRAQ) and metabolic labeling (e.g., SILAC). These approaches have been frequently employed to monitor changes in phosphopeptides and the latter approach, ^15^N metabolic labeling, was recently used to identify the RAPID ALKALINIZATION FACTOR (RALF) peptide receptor in Arabidopsis. The success of metabolic labeling to identify reliable changes in the phosphorylation states of plasma membrane proteins was highlighted by Stes et al.

While the plastid represents a unique structure found in plants, studying this organelle also provides information on the evolution of eukaryotic plants. Since the initial endosymbiotic event, the majority of plastid derived genes were relocated into the nuclear genomes of their host, resulting in the evolution of an N-terminal targeting mechanism in plants. The cleavage of transit peptides during protein import into the plastid can be studied through the application of a sample preparation procedure known as terminal amine labeling of substrates (TAILS). This technique was applied to a freshwater alga (*Cyanophora paradoxa*), a representative of one of the main lineages after endosymbiosis of the ancestral pre-plastid (Köhler et al.). An intriguing finding from this analysis was that low abundant proteins may have evolved an alternative import mechanism in this lineage.

Pollen coat proteins are essential for a range of factors involved in the interaction with the stigma, including adhesion, recognition, hydration and germination. These features are essential requirements for successful reproduction and ultimately the development of seeds and fruits. The pollen coat proteins of maize, an economically important crop, have only had limited attention, with only a handful of proteins (14) characterized to date (Gong et al.). Pollen coat proteins are prepared by initially treating with an organic solvent then extracted using detergent buffers. These approaches have only recently been applied to maize pollen, and given the initial success, this enrichment approach should yield a deeper proteome in the future.

The diversity of secondary metabolites found in plants is immense and is of great interest due to their involvement in the biosynthesis of many essential plants-specific compounds (e.g., lignin). Since the biosynthetic pathways of secondary metabolites can occur across multiple compartments or can be specific to organs and can often represent minor components within the cell, a targeted approach is crucial for their study by proteomic technologies (Martinez-Esteso et al.). As such, proteomic surveys are often conducted on specific plant components, for example trichomes, tomato, or plastids to ensure enrichment of specific pathways. Such targeted approaches necessitate very specific sample preparation procedures for protein extraction and enrichment as well as working with species with limited genomic resources for data matching.

### Data

The access and availability to next generation sequencing technologies has enabled the application of proteomics to a wide variety of plant species. The application of proteomics to these newly assembled genomes and transcriptomes can also provide support to computationally derived gene models. The verification of proteins along with the confident assignment of spectral data can be used to create resources that can provide a framework for a proteome atlas; a resource for a plant species displaying expression and organ-specific profiles. While these types of repositories have been created for reference species such as Arabidopsis (Mann et al., 2013), they are only now emerging for economically important plant species such as the common bean (Zargar et al.).

The ability to more readily generate a reference database through next generation sequencing technologies has opened up proteome analyses on species which would have previously been ignored, even if of great scientific interest. Such an example is *Amborella trichopoda*, a shrub endemic to New Caledonia, thought to represent a sister lineage to flowering plants. The recent completion of its genome has enabled the evolutionary exploration protein families, such as the vacuolar processing enzymes which play important roles during seed maturation (Poncet et al.). Such studies can reveal selection biases on specific protein families in these isolated plant species.

## Plant abiotic stress and proteomics

One of the most widely used applications of proteomics in plant biology is to investigate protein changes under abiotic stresses. These investigations are motivated by the desire to better understand plant adaptation to external stresses, especially in the context of improving agricultural productivity. This includes the application of quantitative proteomics to dissect cell specific responses, the identification of proteins involved in stress, the characterization of post-translational modifications involved in the stress response and protein–protein interactions to identify signaling networks that regulate processes for individual and combinations of stresses (Gong et al.; Hu et al.).

Both wheat and barley are major cereal crops and productivity is greatly affected by both abiotic and biotic stresses. A plethora of proteomic studies have been conducted on these two crop species, many with a focus on attempting to identify markers for stress tolerance, such as reactive oxygen species scavengers (Kosová et al.). Reactive oxygen species can be produced during unfavorable conditions, such as stress, and are often found in the apoplast, a compartment that connects the plant to its environment. Extracellular glutathione is sensitive to reactive oxygen and its presence within the apoplast is regulated via a gamma-glutamyl-transferase (GGT). Recently, the link between the regulation of apoplastic glutathione by GGT and the presence of defense enzymes was determined through a proteomic analysis of a GGT mutant (Masi et al.). Thus, it is conceivable that apoplastic glutathione is a major sensing component of the apoplast, connecting the external environment to the cell.

A popular developmental stage selected for abiotic stress studies is germination and seedling establishment, as this is when plants are most susceptible to disease and changing conditions. With increased temperatures and changing rainfall patterns, examining the effects of drought was a common theme for studies in this Research Topic. Examining plant species that are adapted or resistant to a stress is a common approach, and such a study was carried out with drought exposed Holm oak seedlings, a hot and dry adapted tree of the western Mediterranean region (Simova-Stoilova et al.). The 2-DE survey of root extracts from drought effected seedlings indicated that the seedlings responded by adjusting basic metabolic pathways and mobilizing defense systems to counteract the stress. The assessment of drought and heat stress in crops provides a direct link to mechanisms in agricultural relevant species. Such an approach in maize sought to identify early signaling responses in maize seedlings through a phosphoproteomic survey using iTRAQ technologies (Hu et al.). A myriad of phosphoproteins were detected from a range of functional classes, notably there was a significant overlap in the response of drought and heats stressed seedlings at the level of phosphorylation.

Another developmental stage with much attention in this area is that of seed development and storage. The conditions used for seed storage or seed aging has a major effect on seed viability. Proteomics was used to assess changes in germinating *Brassica napus* proteins after seeds were exposed to a treatment of 40°C and 90% relative humidity (Yin et al.). Elevated levels of peroxiredoxin supported prior work indicating a role for reactive oxygen species in contributing to the seed aging process.

There is an inextricable link between abiotic stresses and hormone response in plants. These interactions have been extensively studied using proteomic technologies in an effort to uncover the resulting hormone induced adaptations. The acquisition of desiccation tolerance is a component of a plants life cycle, namely seed maturation. The maize ABA deficient mutant *viviparous-5* was used to dissect ABA-mediated responses to seed maturation by 2-DE from the embryo and endosperm (Wu et al.). A number of proteins were identified as changing in *vp5* seeds, significantly small heat shock proteins (sHSPs) increased and late embryogenesis abundant (LEA) proteins decreased. These findings suggest that sHSPs may be more loosely regulated by ABA during seed maturation.

The effects of temperature conditioning on seeds can significantly impact plant germination and growth and can greatly affect resultant yields. The emergence of sprouts from garlic can be influenced by low temperature treatments, but depending on the duration, can also effect growth rates and yield. A 2-DE survey of the garlic clove subjected to low temperature revealed major changes to metabolic processes which established a new cellular homeostasis that impacts growth rate, plant weight, and yields (Dufoo-Hurtado et al.).

Seagrasses are found in tropical and temperate environments and have important ecological roles in marine habitats. An important factor effecting their distribution is salinity, however little is known about their adaptation and tolerance mechanisms in the saline marine environment. To explore this adaptation process, the impact of a hypersaline environment on the *Cymodocea nodosa* proteome was assessed using 1D-PAGE and tandem mass spectrometry (Piro et al.). The results indicated that the hypersaline treatments increased glycolytic protein levels and vacuolar components (e.g., Na+/H+-antiporter) to deal with these conditions.

Iron and zinc are essential micronutrients for normal plant growth and development and are predominantly taken up from the soil. Iron deficiency is a major problem due to its requirement in redox reactions associated with photosynthesis and respiration and as an essential co-factor for a range of cellular enzymes. The effects of iron deficiency on respiration and other aspects of plant development have been extensively explored using a myriad of analytical techniques including proteomics (Zargar et al.). However, few studies have documented the effect on the apoplast, the compartment initially likely to detect a deficiency. Interestingly, an analysis of the leaf apoplast proteome of *Beta vulgaris* subjected to iron deficiency revealed few changes, indicting the apoplastic proteome is primed to deal with changes in iron concentrations (Ceballos-Laita et al.). Elevated zinc levels can result in zinc salts in the soil, which produce an osmotic response similar to that of saline stresses. To examine the effect of elevated zinc levels, it is thus important to also examine the effects of salt stress. Lettuce (*Lactuca sativa* L.) is a popular and economically important vegetable crop which is sensitive to salt stress and grown in a variety of soil types. The leaves of lettuce exposed to zinc and salt stress were analyzed by tandem mass spectrometry and revealed an accumulation in proteins related to glycolysis, nitrogen metabolism, hormone biosynthesis and protein metabolism (Lucini and Bernardo). The overlap of proteins identified between elevated zinc and the salt stress were similar, although the zinc stress appeared to enhance the effects.

The ultimate abiotic stress for plants is high levels of ionizing radiation, conditions which exist in the radio-contaminated areas around Chernobyl and Fukushima. Proteomic studies to assess the effects of these environments have been conducted on seeds harvested from soybean and flax from radio-contaminated areas of Chernobyl during two successive generations (Rashydov and Hajduch). The seed proteomes had altered abundances of glycine betaine, seed storage proteins, and proteins associated with carbon assimilation.

## Plant biotic stress and proteomics

Investigating plant pathogen interactions is a major focus in plant biology as it attempts understand this critical and often deleterious interaction. This is often driven by the desire to reduce the impact of disease in crops, which is thought to result in 10–20% reductions in yield per year. Much of the work focuses on determining the biochemical and molecular parameters associated with host resistance or susceptibility. These can often manifest itself as specific resistance traits (e.g., a specific receptor) or general properties (e.g., thickened cell walls). Plant proteomics has played a significant role in extending our knowledge in this area as it attempts to uncover mechanisms of resistance.

The apoplast plays a central role in between plants and pathogens as it represents the initial interaction and communication point during infection. Thus, it is no surprise to find a plethora of proteomic surveys applied to the dissection of this compartment associated with plant pathogen interactions (Gupta et al.). This includes multiple analyses of the secretome from a range of plant species infected with specific pathogens. A specific example is the proteomic analysis of the apoplastic fluid of the *Coffea arabica* in an attempt to identify resistance markers. The coffee industry has been devastated for over a century by coffee leaf rust caused by the fungus *Hemileia vastatrix*. Employing 2-DE in combination with a susceptible and resistant variety of *C*. *arabica*, a range of resistance associated proteins were identified, including glycohydrolases, proteases, and other pathogenesis-related proteins (Guerra-Guimarães et al.).

## Proteomics to profile plant cultivars

The variations in response, growth, and yield of different cultivars has been fundamental in our ability to expand the range and adaptability of crop plants. However, the confident assignment and detection of markers in various cultivars for factors such as pathogen resistance and stress tolerance is challenging. While omic technologies have attempted to play a role in marker discovery and determination between cultivars, these approaches have had varying success. However, they likely represent one of the most promising options for breeders when used effectively (Zivy et al.).

Processes such as cold acclimation can significantly improve the tolerance of a many plants to freezing temperatures. The process of cold acclimation in alfalfa (*Medicago sativa*) is essential to enable tolerance during severe winter months. A proteomic analysis was conducted to uncover the acclimation process in alfalfa of a freezing-tolerant cultivar compared to a freezing-sensitive cultivar (Chen et al.). The analysis revealed that more proteins changed in the resistant cultivar when subjected to cold acclimation supporting the notion of priming of the cultivar for freezing conditions.

The grape industry produces over 75 million metric tons annually. Grapevine production can be significantly affected by drought, climate and salinity. The completion of the grapevine genome in 2007 has enabled a range of proteomic surveys to be confidently conducted on the effects of abiotic stress at the developmental and varietal level (George and Haynes), again demonstrating the wealth of knowledge in genetic varieties. The grape berry skin is rich in secondary metabolites and is an important mechanical barrier against pathogens and damage by injury. Various grape cultivars are known to vary in their anthocyanin content, and thus a metabolomic and proteomic analysis was performed on the grape cultivars Riesling Italico, Pinot Gris, Pinot Noir, and Croatina (Negri et al.). The analysis indicated a relationship between secondary metabolism and pathways associated with primary metabolism in the development of the grape berry skin.

Barley is relatively salt and drought tolerant, however varieties can respond differently. The response of crowns from the barley cultivar Amulet to varying soil water capacities was examined using 2D-DIGE (Vitamvas et al.). A range of metabolic and protective enzymes were found to respond, however physiologically the cultivar was found to be sensitive to drought stress.

## Proteomics and post-transcriptional regulation

The discrepancies observed between transcriptional and protein responses in plants have been amplified as proteomic technologies have advanced. While this difference does not appear to be strongly observed in mammals and yeast, the question arises “Are plants special?” (Velez-Bermudez and Schmidt). The observations that plants have specialized ribosomes with the potential for high levels of heterogeneity and distinct gene splicing mechanisms supports the proposition that plants may have additional controls that explain the observed discordance. A case in point is that of seed germination and aging, where it would appear that translation of stored mRNA is an essential component of seed quality. Thus in many species, factors effecting stored mRNA make a more significant contribution than those effecting transcription. The control of germination is thus likely to constitute mRNA translation and protein post-translational modifications rather than transcriptional regulation (Galland and Rajjou). Therefore, the application of proteomic technologies to seed germination represents an essential tool to dissect this vital aspect of plant growth and development.

## Conclusion

The 1st INPPO World Congress in 2014 marks the initial steps in bringing plant proteomic research to a level currently undertaken in the areas of human and microbial research. The collection of submissions to this Research Topic, to specifically support the inaugural INPPO congress, highlights the range of activities currently being undertaken by plant proteomic researchers and reflects the current state of the field. These submissions highlight the fact that plant proteomics only touches the surface of biological complexity and we are likely far from exploiting the full potential of this technique. At the methodological level, much work still employs first generation approaches (2-DE), but the community is slowly moving to more advanced approaches. There are still important challenges yet to be faced, including revealing more of the proteome, confident protein identifications with quantification, data validation, and addressing orphan and recalcitrant species. Ultimately the community needs to improve its adoption of advanced techniques to enable us to better address biological questions and develop translational outcomes. These questions will be key discussion points during the 2nd INPPO World Congress, due to be held in September of 2016 in Bratislava (Slovakia). It is anticipated that the diversity of approaches and quality of science will meet and likely exceed that of the inaugural INPPO of 2014.

## Author contributions

JH wrote the draft. JJ, GA, SM, and SL edited and contributed to the initial draft. JH compiled the final version.

### Conflict of interest statement

The authors declare that the research was conducted in the absence of any commercial or financial relationships that could be construed as a potential conflict of interest.
